# A Comparative Evaluation of Contact Angle and Depth of Penetration of Sodium Hypochlorite With Various Surfactants: An In Vitro Study

**DOI:** 10.7759/cureus.62480

**Published:** 2024-06-16

**Authors:** Shubhashini N, Krithika D, Akhilesh Gowda, Shruthi Nagaraja, Rhea S Mathew, Nivaskumar G A, Vinaychandra R

**Affiliations:** 1 Department of Conservative Dentistry and Endodontics, Rajarajeswari Dental College and Hospital, Bengaluru, IND; 2 Department of Conservative Dentistry and Endodontics, Faculty of Dental Sciences, Ramaiah University of Applied Sciences, Bengaluru, IND

**Keywords:** endodontics, 2.5% sodium hypochlorite, propylene glycol, confocal laser scanning microscopy, 0.2% cetrimide

## Abstract

Objective: Sodium hypochlorite (NaOCl) is regarded as the most frequently used root canal irrigant. Its high surface tension prevents its penetration into complex canal anatomies. The present study assesses the contact angle and penetration depth of 2.5% NaOCl with 0.2% cetrimide and propylene glycol.

Material and methods: Sixty recently extracted mandibular premolars with a single root were obtained. Thirty were sectioned longitudinally, and the remaining 30 teeth were sectioned transversely. Acrylic blocks were used to mount the parts, and 5 µL of each of the following solutions was placed on the dentin surface: Group 1: 2.5% NaOCl (control), Group 2: 0.2% cetrimide + 2.5% NaOCl, and Group 3: propylene glycol + 2.5% NaOCl. Following this, contact angle analysis was made using a contact angle goniometer. We prepared and instrumented access cavities in 30 teeth to work up to the size of the ProTaper Gold F2 (Dentsply Tulsa Dental Specialties, Tulsa, OK). Samples were allocated to the three groups, and irrigation was done accordingly. They were sectioned at the coronal, middle, and apical thirds and then subjected to confocal laser scanning microscopy. The data were analyzed using a one-way ANOVA and a Tukey multiple comparison test.

Results: Group 2 had the least contact angle (35.20°) and the highest depth of penetration (DOP; 752.409 µm) when compared to Groups 1 and 3. The DOP decreased significantly from the coronal, middle, and apical thirds. No discernible variation in the contact angle was found between the radicular and coronal portions.

Conclusion: 0.2% cetrimide improved the efficiency of 2.5% NaOCl as an irrigant by lowering its contact angle and increasing its DOP.

## Introduction

The morphology of root canals exhibits remarkable complexities that are not completely accessed by instrumentation alone. Chemical debridement through irrigation has an important role to play in reaching these uninstrumented areas and eliminating pathogenic microorganisms. Research indicates that microorganisms can reach up to 2,000 µm into the dentinal tubules [1-3]. Hence, the penetrability of an irrigant is essential to achieving thorough disinfection. Sodium hypochlorite (NaOCl) has been used as an irrigant in endodontics for over a century. It showcases a few desirable properties, like an antibacterial effect and debridement through dissolving organic debris and pulpal tissue remnants. Despite many desirable properties, it falls short in root dentin penetrability due to its high surface tension (48.90 mJ/m^2^) [4].

Wettability is the ability of a solution to spread or adhere to a solid surface. It is measured in terms of the surface tension or contact angle of a solution. A lower contact angle corresponds to higher wettability and, in turn, the penetrability of a solution, thus presenting ideal properties for an endodontic irrigant. Wettability can be improved by the addition of surfactants. The past few decades have witnessed the development of various surface-active agents compatible with NaOCl to improve its wettability [5,6]. One such cationic surfactant is cetrimide, or methyl ammonium bromide, which also exhibits excellent antibacterial properties [7]. Its use in combination with irrigants has demonstrated lower surface tension and better penetrability [8,9]. A similar alcoholic surface-active agent is propylene glycol. Propylene glycol's ability to reduce surface tension has found application as a vehicle for better delivery of intracanal medicaments [10], and it has demonstrated better tissue dissolution and antimicrobial properties when combined with irrigants [11].

Previous studies have shown better wettability and penetration of 5% NaOCl with cetrimide and/or propylene glycol. However, the higher concentration of NaOCl poses a higher risk of periapical toxicity and reduces the fracture strength of root dentin [8,12,13]. Structural changes were noted in the dentinal tubules as deep as 400 µm after the use of 5% NaOCl for 30 minutes [14]. In this regard, very few studies have reported the beneficial effects of the two surfactants with lower NaOCl concentrations, and others have reported contradictory results [15]. Due to the limited evidence and literature available in this regard, the present study aimed to evaluate the contact angle and depth of penetration (DOP) of 2.5% NaOCl with two surfactants: 0.2% cetrimide and propylene glycol.

## Materials and methods

The study involved three groups for testing: Group 1 (control group) with 2.5% NaOCl, Group 2 (cetrimide group) with 2.5% NaOCl + 0.2% cetrimide, and Group 3 (propylene glycol group) with 2.5% NaOCl + propylene glycol.

Thirty recently extracted mandibular premolars with single roots were collected, cleaned, and disinfected using 0.1% thymol, with storage in distilled water to analyze the contact angle. Each tooth was sectioned longitudinally and transversely using a diamond disc to prepare four dentin blocks (two coronal and two radicular samples). A total of 120 dentin blocks were mounted on self-cure acrylic with the dentin surface facing upward. The surfaces were polished with 80, 100, 120, and 150 grit abrasive papers and randomly allocated to the three groups (n = 40 per group). A 5 µL solution from each group was pipetted and placed on the coronal and radicular dentin blocks. The contact angle, formed by the tangent of the liquid drop with the flat dentin surface, was measured using a contact angle goniometer (Ossilla, Sheffield, UK).

Thirty intact single-canal mandibular premolars were collected, disinfected, and stored to analyze the DOP. The root surfaces were coated with nail varnish, and radiographs were taken with a size 10 K-file inserted into the canal to verify the working length, which was kept 1 mm short of the apex. The specimens were allocated to three experimental groups (n = 10 per group), and each root canal was instrumented with ProTaper Gold (Dentsply Tulsa Dental Specialties, Tulsa, OK) rotary files in the sequence of Sx, S1, S2, F1, and F2. Irrigation was performed with 15 mL of the assigned solution for each group. Following this, the samples were rinsed with 0.9% normal saline, stained with rhodamine B (RB) dye, and rinsed again with saline to wash off the dye. Each tooth was then sectioned at 3, 5, and 8 mm from the apex, corresponding to the apical, middle, and coronal thirds of the root canal, respectively. These sections were observed under a confocal laser scanning microscope (CLSM). The DOP of the irrigant was measured by assessing the penetration of RB dye from the center of each disk at the 12, 3, 6, and 9 o'clock positions, with the average of these four positions considered as the final value.

Statistical analysis was performed using the SPSS software (SPSS version 20, IBM, New York, USA). The data were analyzed with one-way ANOVA and the Tukey post hoc test. An alpha value of 0.05 was set to reject the null hypothesis, ensuring that the differences observed were statistically significant. This threshold was used to determine the reliability and significance of the results obtained in the study.

## Results

In both coronal and radicular sections, the mean contact angle was lowest for Group 2, with values of 35.20° and 35.55°, respectively. This was followed by Group 1, in which 67.55° in the coronal section and 67.30° in the radicular section were displayed. The highest mean contact angle was measured in Group 3, with 69.35° in the coronal section and 69.60° in the radicular section (Table 1).

**Table 1:** Comparison of mean contact angle in the coronal and radicular regions between three groups SD: standard deviation

Region	Groups	N	Mean	SD	Min	Max	P value	Sig. diff.	P value
Coronal	Group 1	10	67.55	6.18	57	79	<0.001	G1 vs. G2	<0.001
	Group 2	10	35.2	3.85	26	41		G1 vs. G3	0.62
	Group 3	10	69.35	7.56	53	82		G2 vs. G3	<0.001
Radicular	Group 1	10	67.3	5.33	58	78		G1 vs. G2	<0.001
	Group 2	10	35.55	5.57	26	45		G1 vs. G3	0.35
	Group 3	10	69.6	4.67	63	80		G2 vs. G3	<0.001

**Figure 1:**
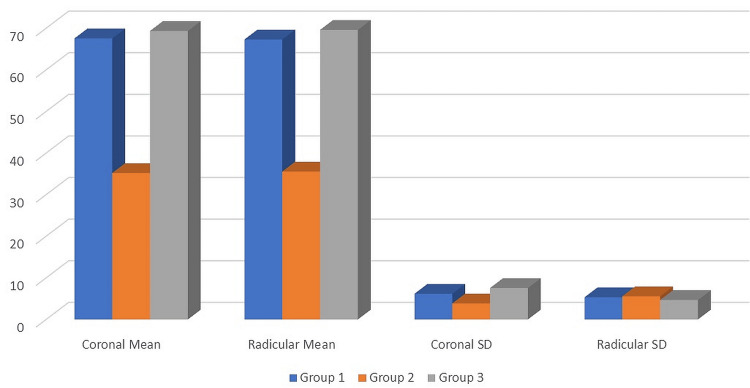
Mean contact angle between coronal and radicular regions in each group

When compared between the groups, Groups 1 and 2 and Groups 2 and 3 displayed notable variations in their average contact angles. Figure 1 shows that there was no significant difference between Groups 1 and 3 when the p value was less than 0.001.

**Figure 2:**
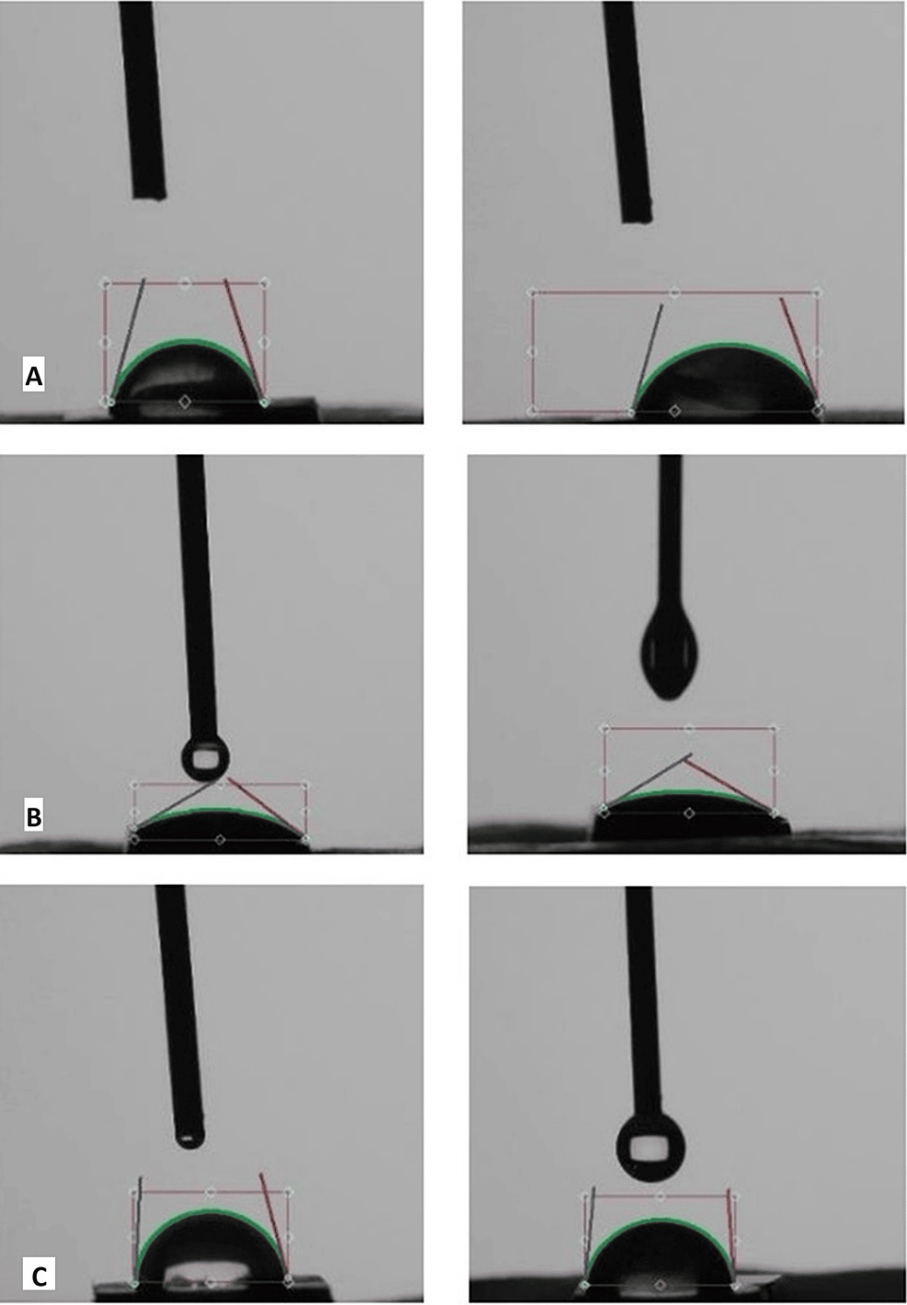
Contact angle in coronal and radicular sections of the root dentin for irrigation with (A) 2.5% NaOCl, (B) 2.5% NaOCl with 0.2% cetrimide, and (C) 2.5% NaOCl with propylene glycol NaOCl: sodium hypochlorite

Contact angle images obtained from the contact angle goniometer are depicted in Figure 2.

In coronal, middle, and apical thirds, Group 2 had the highest mean DOP, with values of 752.409 µm, 707.149 µm, and 599.945 µm, respectively. Group 1 showed intermediate results, with a mean DOP of 633.790 µm in the coronal, 582.944 µm in the middle, and 505.929 µm in the apical third. The least mean DOP was observed in Group 3, with 608.692 µm in the coronal, 568.146 µm in the middle, and 473.554 µm in the apical third (Table 2).

**Table 2:** Comparison of mean DOP (in µm) between three groups in different regions SD: standard deviation; DOP: depth of penetration

Region	Groups	N	Mean	SD	P value	Sig. diff.	P value
Coronal	Group 1	10	633.79	53.102	<0.001	G1 vs. G2	<0.001
	Group 2	10	752.409	48.771		G1 vs. G3	0.56
	Group 3	10	608.692	60.289		G2 vs. G3	<0.001
Middle	Group 1	10	582.944	83.799	<0.001	G1 vs. G2	0.001
	Group 2	10	707.149	53.778		G1 vs. G3	0.87
	Group 3	10	568.146	57.618		G2 vs. G3	<0.001
Apical	Group 1	10	505.929	61.789	<0.001	G1 vs. G2	0.004
	Group 2	10	599.945	70.012		G1 vs. G3	0.46
	Group 3	10	473.554	44.936		G2 vs. G3	<0.001

**Figure 3:**
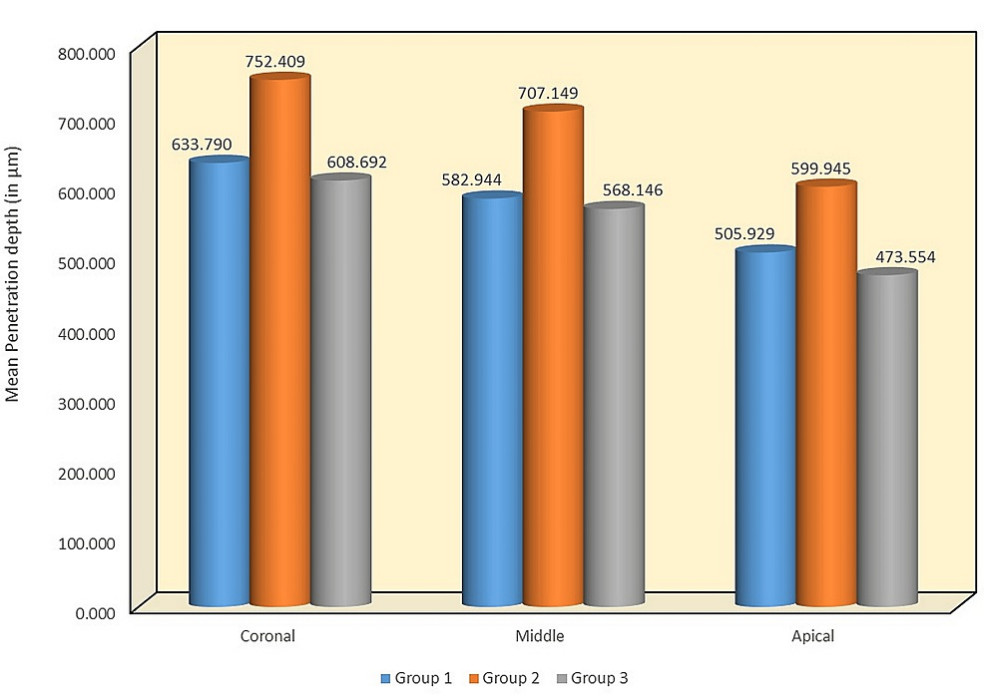
Mean DOP (in µm) between three groups at different regions DOP: depth of penetration

**Figure 4:**
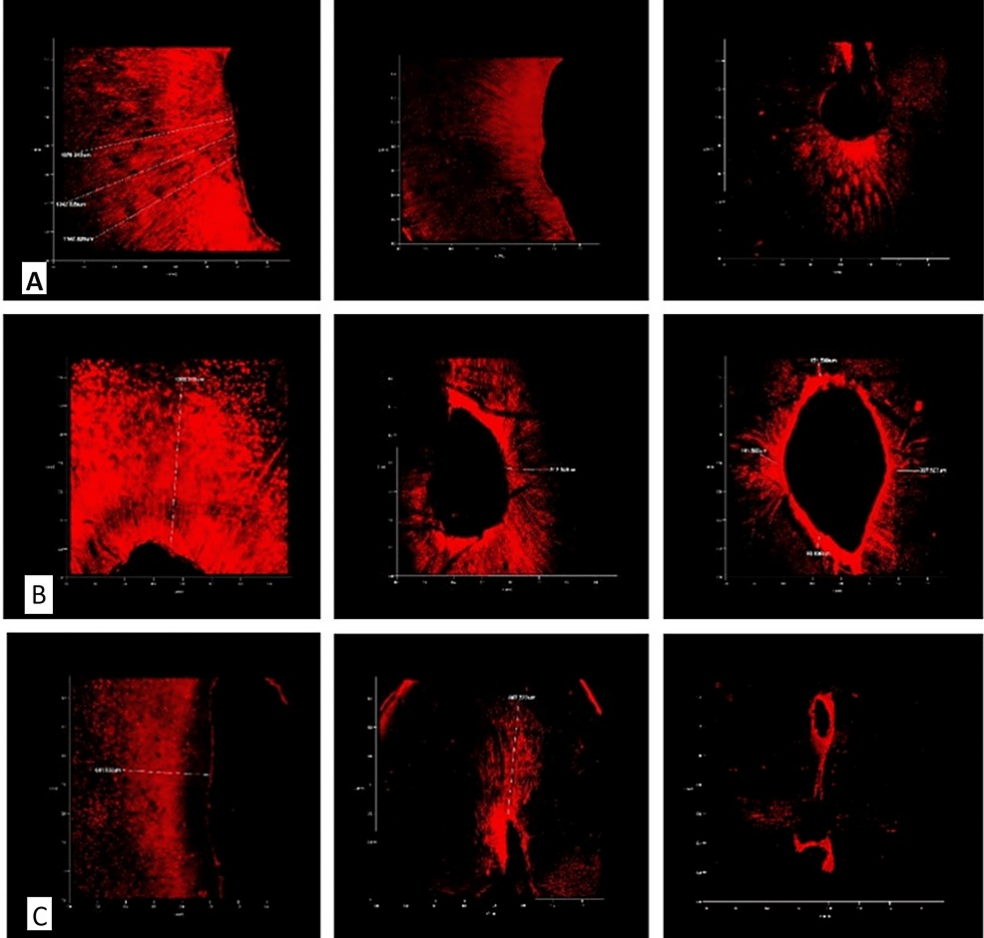
Confocal images of DOP in coronal, middle, and apical sections of the root dentin for irrigation with (A) 2.5% NaOCl, (B) 2.5% NaOCl with 0.2% cetrimide, and (C) 2.5% NaOCl with propylene glycol DOP: depth of penetration; NaOCl: sodium hypochlorite

When compared between the groups, the results were similar to those of contact angle, with notable differences in the average penetration depth between the groups (1 and 2) and (2 and 3). However, Figure 3 shows that there was no significant difference between Groups 1 and 3. Confocal images obtained from CLSM are depicted in Figure 4.

The highest penetration depth was noted in the coronal region, followed by the middle and apical thirds. When the contact angle was analyzed, no significant difference was noted between coronal and radicular samples.

## Discussion

Surfactants act as detergents, emulsifiers, or wetting or foaming agents [16]. When added to the irrigants, these agents reduce their surface tension, enhance microbial effectiveness, and improve clinical performance [17]. Cetrimide is one such surfactant that has shown positive results as an irrigant when evaluated along with NaOCl. The present study used 0.2% cetrimide and propylene glycol as surfactants. When used with 5.25% NaOCl, these two surfactants have shown better wettability and penetrability [8,12]. Studying the use of surfactants with a lower concentration of NaOCl is essential to balance safety and efficacy in endodontic irrigation. Higher NaOCl concentrations pose risks of toxicity and tissue damage, but surfactants like cetrimide and propylene glycol have shown to enhance the wettability and penetrability of NaOCl solutions. By investigating these surfactants at a lower NaOCl concentration, the study aims to determine if they can maintain or improve antimicrobial effectiveness and pulpal dissolution while minimizing adverse effects. This approach could optimize irrigating solutions, providing a safer yet clinically effective alternative for patient care. The risks posed by higher concentrations of NaOCl as an irrigant prompt the use of lower concentrations [13]. Among the various standardized concentrations of NaOCl used, 2.5% makes an optimal irrigating solution by not posing a significant risk of toxicity and, at the same time, having good pulpal dissolution and antibacterial properties [18,19]. Thus, in the current investigation, 2.5% NaOCl was utilized.

The wettability of an irrigant is determined by the contact angle made by the tangent of the solution on a substrate. A contact angle of less than 90° indicates wetting, a contact angle greater than 90° indicates no wetting, and a contact angle of 0° indicates a completely wet surface [17]. Before the contact angle was analyzed, the dentin surface was flattened out using abrasive grit. The importance of this step has been highlighted in previous studies [20,21]. Surface irregularities that might influence the contact angle formed are thus removed.

All of the study's irrigant groups showed a contact angle of <90°, indicating wetting ability. The cetrimide group formed the least mean contact angle (Group 2). Giardino et al. showed superior wettability of Hypoclean (5.25% NaOCl, propylene glycol, and cetrimide) and Chlor-Xtra as they displayed a 0° contact angle when tested on dentin substrates [19]. Another study that evaluated a modified low-concentration NaOCl (ChlorCid^TM^ Surf containing 3% NaOCl) with surfactant demonstrated lower surface tension, a lower contact angle, and better DOP. They also showed that the solution could maintain a higher alkaline pH and exhibited a similar FAC as ChlorCidTM (3% NaOCl) [22]. No difference between the coronal and radicular sections was observed with respect to contact angle. This may be attributed to a similar processing method involved in the preparation of dentin substrate for contact angle analysis.

DOP was analyzed to determine the efficiency of irrigants in reaching inaccessible areas of the root canal system. The CLSM used in the current study is better suited than other microscopy by producing fewer artifacts. It enables a comprehensive analysis of the sample surface at a smaller magnification. Additional sample preparation is not required; hence, the images are obtained by avoiding sample destruction [23]. RB dye is an ideal dye because of its easier visibility, enhanced infusibility into dentinal tubules, and smaller particle size [24]. In all three groups, the study's results showed that penetration depths were greatest in the coronal thirds, and middle and lowest in the apical thirds. This can be explained by anatomical complexities in the apical third that influence irrigant penetration into the dentinal tubules. Lower permeability is associated with tubular sclerosis, smaller tubule diameters, and fewer tubules overall in the apical thirds [25]. In the current study, Group 2 (2.5% NaOCl+cetrimide) showed the greatest mean DOP in all thirds of root canals. Palazzi et al. measured the surface tension of Hypoclean A and B (5.25% NaOCl with cetrimide and polypropylene glycol as two surfactants) and discovered it to be much lesser than 5.25% NaOCl and Chlor-Xtra (6% NaOCl), indicating better wettability and intratubular penetration of Hypoclean [8]. On the contrary, Faria et al. concluded that there was no significant improvement in DOP when 0.2% cetrimide was added to 2.5% NaOCl [15]. The results of the current study reported the lowest mean DOP for Group 3 (2.5% NaOCl+propylene glycol). But, when compared to Group 1 (2.5% NaOCl), the variation lacked statistical significance. Propylene glycol’s alcoholic character could be the reason for this. According to Cunningham et al., adding an alcoholic surfactant may reduce the stability of NaOCl preparations. Within 15 minutes, there was a total decrease in free available chlorine (FAC) when 50% ethanol was added to 2% NaOCl and a 70% loss of FAC in 30 minutes when 30% ethanol was added [26]. However, Poggio et al. demonstrated that TetraClean, a NaOCl-based irrigant containing surfactant polypropylene glycol (an addition polymer of propylene glycol and water), was more effective in removing the smear layer than 17% ethylenediaminetetraacetic acid [27].

The limitations of the study are that no methods of irrigation activation were attempted to check for an increase in the penetration depth of the irrigant. Also, the effect of surfactant addition on antimicrobial effectiveness, free available chlorine (FAC), and irrigation stability was not analyzed. The above-mentioned shortcomings present a potential scope for future research.

## Conclusions

In light of the study’s limitations, incorporating 0.2% cetrimide improved the wettability of 2.5% NaOCl by reducing the contact angle and increasing the penetration depth. No significant difference was observed with the addition of propylene glycol. A simple addition of a surface-active agent proved to be beneficial in improving the efficiency of root canal disinfection, even at lower concentrations of NaOCl. Thus, it can be possible to avert potential risks posed by a higher concentration of NaOCl.
